# How to Perform a Post-Morterm Examination: The Experience of a Lifetime, and Its Practical Teaching

**Published:** 1907-10-05

**Authors:** Henry D. Littlejohn

**Affiliations:** late Professor of Forensic Medicine University of Edinburgh.


### Hospital Clinics.

### HOW TO PERFORM A POST-MORTEM EXAMINATION:

### The Experience of a Lifetime, and its Practical Teaching.

An address delivered at the Medical Graduates' College and Polyclinic, London,

By SIR HENRY D. LITTLEJOHN, M.D., LL.D.(Edin.), late Professor of Forensic Medicine University of Edinburgh.

Gentlemen,—For upwards of 50 years I have been lecturing to medical students, and this is the first time I have had the opportunity and the pleasure of addressing medical practitioners. I can assure you I esteem it a very great honour indeed. I am only sorry that what I am about to give you is, possibly, not more worthy of your attention.

I shall to-day look back on the records of my life, and review the pitfalls into which I have fallen, so as to guide you against falling into them in your own careers; and also to give you, as general practitioners, hints as guides in your forensic work. We cannot always have an expert at our elbow, and the general practitioner who is first called in to a case, trained as he is to habits of observation, sees what others may readily overlook. You, gentlemen, notice the things which are of the most vital importance. Never have I forgotten that case which occurred during the later years of Napoleon III.'s Empire, when there were certain mysterious movements in the air forecasting the downfall about to come. Prince Bonaparte quarrelled with a musical critic, sounds of firearms were heard, and when people rushed in they found that the poor journalist had been shot. They sent for a practitioner who lived five or six houses off. He came, and was the first to see—what? The position of the furniture in the room, certain changes and marks upon the furniture, and, therefore, he could give most important information. He was passed over, and M. Tardieu and Bergeron made the post-mortem examination. They never thought of calling in the practitioner; he was beneath them. The case came to trial, and, to everyone's astonishment, the medical practitioner was called for the defence, and overthrew all the evidence of the experts. I beg of you to remember that you are not experts, but men of observation, who can see things which are of vital importance in the detection of crime. You remember what happened to a greater man than any of us, Sir Astley Cooper. He was summoned to Deptford to see a Mr. Rose, who had been shot in his office. It was in the days of coach-horses, and Cooper drove rapidly down, but Rose was dead when he arrived. He had to wait to get fresh horses to come back to London; and they said, "Do you care to go into the office to see where this poor man was sitting?" He said, "I have nothing to do for half an hour; I will go." Then he said, "What side was he shot on?" He then hazarded the opinion that he thought the man who fired the shot must have been left-handed. There was a partner of this Mr. Rose, of the name of Patch; he had been a most intimate friend of Mr. Rose and appeared to be the last man in the world to be guilty of the crime. But it happened that various facts began to come out, and Patch was examined. He was handed the Book to kiss, and he put out his left hand for it. That drew attention to Sir Astley Cooper's remark. Finally, Mr. Patch was tried, and executed for the murder of his partner. Many similar cases have occurred in my own practice.

### The Post-Mortem Examination.

I shall now give you one or two hints with regard to the examination of dead bodies.

When is the post-mortem to take place? It was only on Friday last that the Editor of "Chambers's Encyclopædia" came over to my office and produced a leaf of his encyclopædia, and showed me a little article which he had put in many years ago, in which the man who wrote it stated," "No post-mortem should be performed until after the expiry of 24 hours from the time of death," and the Editor said, "What is your experience?" The Editor was a gentleman from the Midland counties, and a doctor had written to ask whether there was any legal authority for that statement, and the Editor came to ask me. I said, "There is no legal authority, at least I know of none, either in England or Scotland, but I may tell you what are the regulations of our great hospital, the Royal Infirmary of Edinburgh. They allow a post-mortem, if deemed advisable, to take place within 10 minutes or 15 minutes of death; and the relatives can remove a body from the infirmary in as short a time." I remember that certain hospitals in England have made the by-law that no body should be removed until after the expiry of 24 hours. I think that was to give the medical staff an opportunity of bargaining with the friends as to a post-mortem examination. Two medical men ought to conduct every post-mortem examination made in connection with legal proceedings. It is a scandal to find, in important trials, when a man's life, remember, is dependent on the medical opinion, that only one inspector was present at the post-mortem examination. There must be two. It is a perfect pleasure to me to have a plain, ordinary educated practitioner to be my colleague. He can always say, "I saw this; or I saw that." He may not be able to fence with a barrister so well as an expert, but I have the satisfaction in giving evidence that I know it can be corroborated by an honest-minded practitioner. One examiner must take notes while the other performs the examination. "Who is to draw up the report?" One of the two must do it, and the two must agree on the terms of the report. "Who shall sign first?" These petty questions occur. I have the easiest way out; look at the register and find who is the senior, and let him sign first. I remember a well-known "chestnut" with regard to a Highland chieftain. His foster-brother, who acted as a kind of servant to him, said, "Well, chief, at the banquet I did not think you had your proper position." "Oh," said the chief, "Macdonald, wherever I sit, that is the head of the table." The first step is to make an external examination. If you turn a dead body over a quantity of fluid escapes from the mouth; turn the body back, and that fluid passes into the air-passages, and then when you come to examine the air-passages you coolly report that you found a quantity of fluid there. Who put it there? Yourself. Therefore you must keep the body on the back until you have finished the examination.

### The Importance of Body Marks.

We observe the post-mortem rigidity and lividity, and, of course, we look at any suspicious mark. I have the honour of examining for the Fellowship of the Royal College of Surgeons of Edinburgh, and my subject is forensic medicine. I have intelligent men before me, and I ask them, "If you find a suspicious mark upon a dead body, what is your duty?" "Our duty is plain, we incise it." "Right. Why?" "In order to distinguish it from a mother's mark." "You have answered remarkably well." Now comes the question, "What other important lesson have you gained from making that incision? You have made out there is effusion of blood, and that that must have been the result of a bruise. What is the other important lesson?" I seldom get an answer. It is to make out the AGE of the bruise. This may be of vital importance to a person charged with crime. A commercial traveller has a drunken wife. He comes home, after a week's work, finds the woman lying drunk at home, with no food, and the children crying. The neighbours say they heard violent language, they heard one or two falls, and they thought they heard something like blows. A doctor examines the body, and says, "Ah, yes, bruises. Dear me; these are most suspicious." How important, now, it is, gentlemen, that you should make out the age of those bruises. Here is a drunken woman tumbling about that house for days, falling against the fender, falling against this chair and that, and though there are bruises, not one of them is of recent date. If you report in such a case that death is believed to be due to violence, the poor fellow is apprehended, an examination is made, and the doctor says, "There is no doubt, my lord, that the bruises on this woman's body were probably sufficient to account for death." I have had the great pleasure, in the course of my long experience, of rescuing a man like that from this very foul imputation. Whenever there are marks of violence on projecting parts of the body you must allow in the witness-box that they might have been produced by a fall. It will not do for you, when in the witness-box, when a man's life is hanging on your evidence, to ask whether you need bother about this. I say yes, allow that all injuries at the back of the head, on the shoulders, hips, and other projecting parts of the body might have been the result of a fall. Now, you see the importance of estimating the age of a bruise. Of course, if we find bruises on certain parts of the body, such as the arm-pit, and other places where they could not be accounted for by falls, these throw a lurid light on the causation of the other bruises.

### Where to Start.

Now, gentlemen, you have got a knife in your hand, the body is before you; how shall you commence? It is very hard for me to contradict such a man as Virchow, who, in his celebrated book upon post-mortem examinations, says: Commence with the head. I say nothing of the kind. The first thing to be observed is the condition of the heart, so far as its fulness or otherwise is concerned. Where will you begin? Two inches above the pubes. Make your incision very carefully. I then put in my fingers, and pull up the abdominal parietes, and with the thick border of my knife downwards I incise, always avoiding the distended intestines. In 90 per cent. of cases of sudden death there is an enormous distension of the stomach. To penetrate the stomach, especially if there are any persons watching you—possibly solicitors—is unfortunate, and if it is a case of suspected poisoning you lose some of the contents of the stomach, and you may possibly have to confess that in the witness-box. In cutting upwards towards the surface in this way you save all injury to the stomach, liver, and intestines. Then make an incision from the sternum to the ensiform cartilage, and divide the attachments of the diaphragm on either side. Pass in your hand and break up the adhesions in the mediastinum. You may find an aneurism, and, to people's astonishment, you say, "I think the cause of death has been made out." If there is no aneurism you cut through the ribs at their attachment to the sternum. If in doing so you find, in a man of 40, ossification well commenced, you have learnt something. Don't cut through the sterno-clavicular joint and remove the sternum. Leave the upper portion of the sternum intact, so that when the friends see the body they do not observe the deep sulcus which would otherwise be left. In that case they may think you have removed half the interior of the chest.

### The Heart and Lungs.

Now examine the heart and lungs. The heart is the first organ to be examined, therefore I incise the pericardium, and I see marked distension of the right side of the heart, and I see that the left side is contracted like a cricket-ball. I do not open the heart, because if I did it would spoil my dissection, but I put my fingers on the right auricle and on the right ventricle, and I can tell, by mere palpation, the character of the blood—whether it is fluid or coagulated. You cannot step into a witness-box and speak of a case as one of asphyxia unless you can say the blood was fluid. Having done that, I can follow Virchow's directions, and proceed to examine the head. Have you ever attended a post-mortem examination in the pathological theatre of any of the hospitals? They always begin with the head. They take out the brain, and the head falls over the end of the table, and there is a gush of blood. It has come from the right auricle and ventricle, and the report afterwards says the heart was found empty. Think of the great Taylor reporting recent cases of death from drowning and saying, "I have got it from the practitioners themselves that the heart was empty." I can quite understand that, when they begin with the head, according to the rules in the books, and do not examine the heart first of all.

### The Stomach and Intestines.

If I were asked by the Crown to be present and watch the results of a post-mortem examination, I should be pleased if the gentleman in charge instantly ligatured the stomach and put it aside for special separate examination. With your sharp knife you are apt to injure it; and if you pull away the liver and the intestines you are apt to force the contents of the stomach upwards into the œsophagus or down into the duodenum. You can judge of the effect of that in any case of poisoning. Think of the comedy which was carried on at the celebrated post-mortem examination on John Parsons Cook, poisoned by Palmer. They gave the stomach to the oldest gentleman there. It was opened, and Palmer took care to cannon up against him, and the contents of the stomach were spilled. That accounts for the remarkable fact that Taylor, with all his skill, failed to detect any trace of strychnine in the stomach of John Parsons Cook.

We now examine the head, making the incision from ear to ear. You may think I am romancing when I tell you that I think I have made eight post-mortems conducted by myself and the Army authorities at Edinburgh when they had already begun the dissection. The army surgeons made the incision from the forehead to the back of the head. Think of the mark when the friends of the poor soldier come for the body. Now we use the saw. I do not know of a more trying thing than to remove the skull-cap in the presence of a lot of people who are watching you. Think what the great Liston did. There was a large tenement house to be taken down. He went to the contractors of the buildings, and said, "I should very much like to saw the rafters for you into any length you please." It took him a whole week. But he was a tall, fine fellow, and it gave him an unrivalled facility with his saw, and that was one of the things which made him famous. Contrast him with his great rival—an equally great man—Syme. It was an opprobrium to surgery to watch Syme saw. We thought every awkward harsh movement of the saw involved intense pain, and we hissed. Symes looked up to see who hissed. He went on until we hissed again!

### What You May Learn by Removing the Skull-Cap.

If I were asked what is the perfection in opening the skull, I should say to saw the external table all the way round, and as you have a brittle internal table, by putting the chisel in, crack the internal table round, and then show that the brain is untouched. I cannot do that. I always injure the membranes or the brain a little. But even in taking the skull-cap off we learn something. We have a very large Irish population in Edinburgh, amounting to some 40,000, and they are somewhat turbulent at times; and when I have examined some cases from the Irish quarter I have found that the scalp comes away only with great difficulty, because there are the remains of former bruises and wounds. Again and again I have noted that. In removing the skull-cap you also notice something else. The dura mater is remarkably adherent to the bone. I hold that that shows an alcoholic tendency. As you tear it off you should note this, and it should go into your report. Having removed the skull-cap, incise the dura mater. Then what do we see? We are at once attracted by the appearance of the arachnoid. We first notice the opacity of the arachnoid, secondly, a large amount of sub-arachnoid effusion, and, lastly, those delicate little adhesions between the two sides of the arachnoid. I shall show you that, in the witness-box, you cannot speak with certainty about the alcoholic history of any man unless these points are specially attended to. Before applying the saw, I always pay great attention to the condition of the temporal muscle; and if I find it infiltrated with blood, I am of opinion that the injury causing it has been severe. Now cut through the membranes, and before doing so note the adhesion of the dura mater and such like points.

### The Brain.

How shall the brain be removed? Shall it be *en masse*, or sliced down horizontally? If you remove the brain *en masse*, you lose the fluid from the lateral ventricles; and in making out whether a person has been in a debauch shortly before death it is important to preserve the ventricular fluid to enable the alcohol to be detected. Slice it horizontally and look carefully for any minute aneurisms: look especially at the condition of the cerebral vessels, as to whether they are atheromatous or not. Then examine the base. Strip the membranes off completely, and carefully smell the brain and its cavity. Again and again people have written to ask me how they shall describe the various parts of the brain. They ask, "If they tackle me in the witness-box about the pineal gland, how shall I get out of it?" My reply is: Say the "brain and its appendages" were normal. A barrister, eager to make a point, says, "I do not see that this has been mentioned in your report, doctor." I say, "Oh, you are mistaken, it has." "Well, here is your report; I shall be exceedingly obliged if you will point out the part relating to this portion of the brain which you say you have mentioned." "The brain and appendages were normal"; that includes everything connected with it.

Then there are effusions of blood. An axiom in medical jurisprudence is, "Any blood effused between the dura mater and the bone must be due to external violence." If you find blood there, the middle meningeal artery must have given way, there must be a slight fracture running across the track of the artery.

In your pathological examinations you use a mallet; after sawing through the outer table the skull is struck and you hear a decided crack, showing that the internal table has definitely given way. A professor I know made this mistake. He used a mallet, and the barrister, who had got a hint as to this, procured a mallet, and he said to the professor, "You describe, sir, in your report, this fissure. You used a mallet, didn't you?" "That is the ordinary thing used in our pathological theatre." "Indeed. Is this something like the instrument which you used?" "Now, gentlemen of the jury, here is a gentleman speaking of hair-like fissures. I say that that man produced those fissures by that awful instrument." Blood effused into the tissue of the brain is, as you know, apoplexy, and it is essential that you should be able to speak of the condition of the vessels.

### The Thoracic Viscera.

The chest has already been opened, and we now open the pericardium and examine the heart more carefully. If the pericardium contains blood there must be rupture of an aneurism. Remember that an aneurism may burst into the pericardium by a very minute opening, and unless you are very careful not to remove the heart until you make out that small opening, you are apt to do what has been done repeatedly—namely, make an incision that implicates the minute opening, and the case is described as a wonderful instance of effusion of blood into the pericardium, but how it got there no one can tell. Remember, you must not remove the heart until you have made out the minute opening into the aneurism. It is very common to have an aneurism bursting into the pericardium. If you see a bluish condition of the pericardium, the cause of death is plain, for it is rupture of an aneurism into the pericardium. I open the pericardium, and there is a large clot covering the heart, together with a quantity of serum. A member of our profession, of the name of Stroud, published a first-rate book on the physical cause of our Saviour's death; and I ask you, as a favour to myself, when you see that book on a bookstall, to purchase it. You will find the author makes out most completely that our Saviour's heart must have given way, because when the Roman soldier's spear entered the left side of the chest, out came both blood and water. That is exactly what I have found again and again; a clot and serum. Therefore, when you go to the Continent and see some of those scenes of the Crucifixion, you will be amused to see that sometimes the artist makes the spear enter the right side of the body. It must have been the left side, because there the pericardium was entered, and blood and serum came away.

Examine, of course, the competency of the valves of the heart; pay great attention to that. Watch the mouth of the coronary arteries; with a pair of blunt-pointed scissors cut up their track and see if there is atheroma. It is nonsense to bother about the sudden death of a person if we find the coronary arteries involved, because with that involvement we have a marked impairment of the nutrition of the tissues of the heart.

Now we come to the lungs. Remember that when the blood is fluid it gravitates, and, therefore, when you examine the lungs you see what is called hypostatic congestion, which has been produced by gravitation. It is easy to distinguish between the mechanical congestion of the lungs in death by drowning and the congestion due to bronchitis, incipient pneumonia, etc. One point I would impress upon you. Having taken out the lungs, pass your hands on both sides of the chest in order to discover whether there is a fracture of the ribs. If a man has one, or two ribs fractured, and rigidity has set in, you must put your hand in and press downwards upon the ribs, so as to make out the fractured ends. *(To be continued.)*

